# Scientific Publishing Credibility: Analysis of the Main Factors Threatening It

**DOI:** 10.3390/tomography12010001

**Published:** 2025-12-22

**Authors:** Emilio Quaia

**Affiliations:** Department of Radiology, University of Padova, Via Giustiniani 2, 35128 Padova, Italy; emilio.quaia@unipd.it; Tel.: +39-049-8212375

The scientific publishing crisis represents a complex problem, mainly stemming from the “publish or perish” culture that prioritizes quantity over quality, which leads to the proliferation of low-quality research manuscripts and research misconduct, including data fabrication (making up data or results), falsification (manipulating research materials, equipment, or processes, or changing or omitting data or results such that the research is not accurately represented in the research record), or even plagiarism (the appropriation of another person’s ideas, processes, results, or words without giving appropriate credit) [1]. Other key causes of the scientific publishing crisis include the poor reproducibility of results, with increased frequency of research paper retraction, and the recent threat of fake AI-generated papers, which may also pose the risk of data fabrication and plagiarism. In fact, AI should only be used for tasks like language enhancement, grammar checks, and generating initial drafts; although, AI systems can reuse text and may inadvertently cause plagiarism, raising severe ethical concerns. These factors have eroded the credibility of scientific literature and hindered progress.

What are the causes of this crisis?

(1) I would allocate in the first place the “publish or perish culture”, the “run towards publication”, or even the “obsession of publishing performance”. These terms describe the intense pressure on academics to publish research frequently to secure and maintain their careers, especially for future tenure and promotions. This culture, driven also by factors like university rankings and a focus on quantifiable metrics, can lead to negative consequences, such as prioritizing quantity over quality, performance stress, and a higher risk of unethical practices like data fabrication or predatory journal use.
(2) As a consequence of “publish or perish culture”, I would put in the second place the frequent poor reproducibility of research paper results, leading to paper retraction. A research paper retraction represents the official withdrawal of a published article from the scientific record due to major issues like scientific misconduct, plagiarism, or significant errors in the data. This is often linked to the pressure to publish positive findings, while negative results are often not published. The original paper is typically preserved, but a retraction notice is published to alert readers that the findings are no longer trustworthy and to maintain the integrity of the scientific literature. Many studies claim a significant result, but their findings cannot be reproduced, and this is frequently observed even in high-impact-factor journals. The inability for many published results to be reproduced is a major concern, with a 2016 survey [2] finding that over 70% of researchers could not reproduce another scientist’s experiment. In recent years, the number of retractions has been rising sharply. While the absolute number of retractions has increased significantly in recent years, the rate of retractions has also risen, with approximately 1 in 500 papers retracted in 2023 compared to 1 in 5000 in 2002. This increase is also partly due to improved detection methods, such as new digital tools and a greater focus on research integrity. However, this general increase in research paper retraction has limited the general credibility of scientific publishing.
(3) Rise in predatory journals and AI fraud. Organized networks and, more recently, AI have been used to insert fake papers into the scholarly record, often with fabricated data. The peer review process has been overwhelmed and unable to distinguish fake from real research, although new AI checker software tools may limit this worrying phenomenon, but ths risks producing a breach in the confidentiality of the review process.
(4) High costs and access issues. Traditional subscription journals are expensive, making crucial research inaccessible to many individuals, especially in developing countries. The relationship between high access costs to scientific research and the publication crisis is direct. The escalation of journal subscriptions makes access to research unsustainable for libraries, forcing cancelation, while the pressure to publish produces an increasing number of papers that are not accessed by many studies. Consequently, the results of research are not known by many individuals who could propose other research points or may provide different research ideas or suggestions. Open-access journals may represent a solution to this issue [3].
(5) Honorary authorship corresponding to the intentional misrepresentation of credit to an individual whose contributions to a biomedical article do not meet the criteria for authorship established by the International Committee of Medical Journal Editors (ICMJE) [4] represents a further worrying element that decreases the credibility of scientific research.

What are the real consequences of this crisis?

(1) Erosion of trust: The prevalence of low-quality and fake papers has reduced the overall credibility of the scientific record.
(2) Hindered scientific progress: The scientific publishing crisis can slow down scientific advancement by making it difficult to find and trust reliable information.
(3) Impact on real-world outcomes: In fields like global health, the reliability of research is critical, and a crisis of trust can have serious real-world consequences.
(4) Challenges for legal systems: The decline of the peer-review process as a reliable indicator of the reliability of scientific research raises concerns for legal systems that use scientific evidence.

Are there any potential solutions to the progressive decline of scientific research credibility?

(1) Open access publishing [3]: Shifting to an open-access model can help balance market forces and increase accessibility to research. Unfortunately, even open-access publishing presents several risks, including predatory journals, although the general accessibility to the results of scientific research represents an initial step towards scientific research credibility.
(2) Improved peer-review process: Reviewers are often too busy or may no longer appreciate being involved in the review process, which makes them decline to participate. For this reason, training peer reviewers professionally and providing them remuneration could help improve the quality of peer review. Moreover, the best reviewers should have the highest visibility, and a public database with dedicated scores (e.g., number of reviewed papers and scores) should provide the best reviewer names, similarly to databases that provide authors’ bibliometric scores.
(3) Decentralized science platforms not strictly related to scientific papers: Decentralized Science (DeSci) is an emerging movement that applies blockchain principles to scientific research. It aims to make funding, collaboration, and data sharing more open and transparent. New platforms are emerging that aim to create more efficient and collaborative ways to share research.
(4) Changes in academic incentives: Shifting the focus from quantity to quality in academic evaluation could help address the root cause of the “publish or perish” culture.
(5) Increase the weight of parameters other than publishing of scientific papers to increase scientific ranking and academic promotion, including patents and the capability of a researcher as principal investigator in attracting grants, which should be considered at the same level or even above the number of publications and publication metrics.
(6) More consideration for new bibliometric indices, including Eigenfactor in journal ranking [5] and Crown indicator [6] in author ranking: Eigenfactor, developed at Washington University, measures the number of times articles from the journal published in the past five years have been cited in the Journal Citation Reports (JCR). Like the impact factor, the Eigenfactor score is essentially a ratio of the number of citations to the total number of articles. However, unlike the Impact Factor, the Eigenfactor score counts citations to journals in both the sciences and social sciences, eliminates self-citations, discounts every reference from one article in a journal to another article from the same journal, and weights each reference according to a stochastic measure of the amount of time researchers spend reading the journal. The Eigenfactor uses Thomson Reuters Web of Science citation data, and Eigenfactor scores are scaled so that the sum of the Eigenfactor scores of all journals listed in Thomson’s Journal Citation Reports (JCRs) is equal to 100. Crown indicator [6] represents a much-studied bibliometric indicator that normalizes citation counts across fields. Both these indices may overcome the impact factor and H index limitations [7] and could provide more credibility to scientific research, which should be based on a single research paper’s validity rather than on journal reputation and impact factor.
